# Analyzing efficiency of a lentiviral shRNA knockdown system in human enteroids using western blot and flow cytometry

**DOI:** 10.1016/j.xpro.2024.103082

**Published:** 2024-05-22

**Authors:** Adam P. Wilson, Karni S. Moshal, Addison P. Franca, Sasirekha Ramani, Randle Gallucci, Hala Chaaban, Kathryn Y. Burge

**Affiliations:** 1 Pediatrics, University of Oklahoma Health Sciences Center, Oklahoma City, OK 73104, USA; 2 Molecular Virology and Microbiology, Baylor College of Medicine, Houston, TX 77030, USA; 3 Pharmaceutical Sciences, University of Oklahoma Health Sciences Center, Oklahoma City, OK 73104, USA

**Keywords:** cell culture, flow cytometry, gene expression, protein expression and purification, cell differentiation, organoids

## Abstract

Enteroids are *in vitro* models to study gastrointestinal pathologies and test personalized therapeutics; however, the inherent complexity of enteroids often renders standard gene editing approaches ineffective. Here, we introduce a refined lentiviral transfection protocol, ensuring sufficient lentiviral engagement with enteroids while considering spatiotemporal growth variability throughout the extracellular matrix. Additionally, we highlight a selection process for transduced cells, introduce a protocol to accurately measure transduction efficiency, and explore methodologies to gauge effects of gene knockdown on biological processes.

## Highlights

• Protocol for lentivirus-based gene editing of gastrointestinal enteroids
• Steps for performing a puromycin selection trial
• Instructions for assessing transduction efficiency

## Abstract (editor highlights)

Publisher’s note: Undertaking any experimental protocol requires adherence to local institutional guidelines for laboratory safety and ethics.

## Graphical abstract

**Figure:**
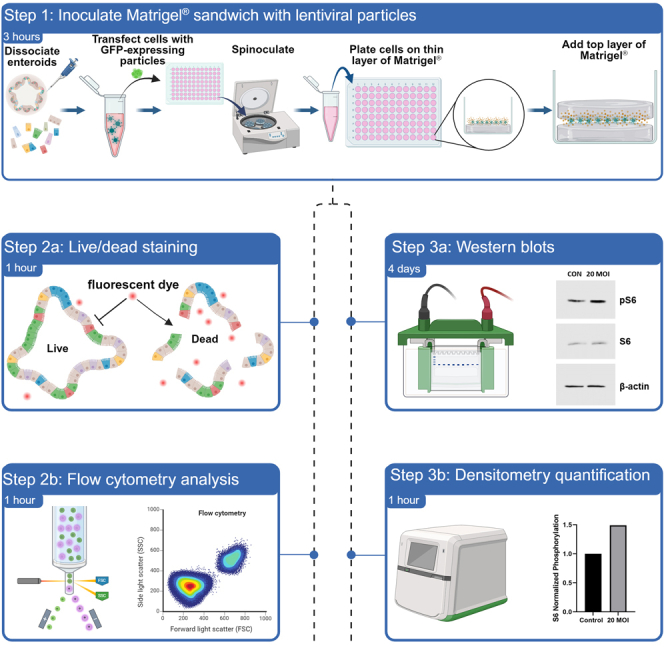

## Before you begin

The protocol below describes the lentiviral transfection of human preterm ileal enteroids with the objective of increasing mechanistic target of rapamycin complex 1 (mTORC1) activation through gene knockdown of its negative regulator, tuberous sclerosis complex 2 (*TSC2*).^1^ Enteroids provide a highly translational model of the human intestine.^2^^,^^3^^,^^4^ While various protocols have been described to genetically modify enteroids,^5^^,^^6^^,^^7^^,^^8^^,^^9^^,^^10^^,^^11^^,^^12^^,^^13^ technical challenges have often limited efficacy of genetic manipulation in these cultures.^14^^,^^15^^,^^16^ Here, we introduce a procedure predicated upon methodology from Maru et al.^17^ This protocol incorporates a puromycin selection cytotoxicity trial, preparation of enteroid media, lentiviral transfection of enteroids, and flow cytometry, electrophoresis, western blotting, and densitometry to assess transfection outcomes. As this protocol involves the use of lentiviral particles, all steps are performed within a type II biological safety cabinet in a biological safety level 2 (BSL2) laboratory.

### Institutional permissions

Ileal tissue was collected from preterm infants undergoing gastrointestinal surgery following approval by the University of Texas Health Science Center at Houston (UTHSC) Institutional Review Board (IRB) and parental consent. Infant tissue samples were transferred to Baylor College of Medicine under a material transfer agreement (MTA) for establishment of enteroids. Enteroids (Line IL1004, see key resources table) were generated at the Texas Medical Center Digestive Diseases Center (DDC) Gastrointestinal Experimental Model Systems (GEMS) Core from intestinal crypts isolated from the surgical tissues of infants.^18^^,^^19^^,^^20^ The enteroids were then transferred to the University of Oklahoma Health Sciences Center (OUHSC) under an MTA.

### Puromycin selection cytotoxicity trial

**Timing: 9.5 days**

Viral vectors for transfection typically incorporate an antibiotic-resistance gene, such that transfected mammalian cells can be selected for by introduction of sufficient concentration of the antibiotic. Each cell line differs with respect to the antibiotic concentration required for appropriate selection, however. Therefore, a titration experiment is required to determine the lowest concentration of antibiotic needed to both eliminate uninfected cells and limit off-target effects or unnecessary cytotoxicity in transfected cells.^21^ Here, we use particles produced by the pLKO.1 vector, which transfers puromycin resistance to transfected cells. Typically, 1–10 μg/mL is sufficient to kill most uninfected mammalian cell types. Because enteroids present additional technical challenges, our laboratory has also trialed puromycin concentrations above 10 μg/mL.

This step dissociates enteroids into a single-cell suspension for Matrigel sandwich plating on a 96-well microplate. Because lentiviral particle penetrance of Matrigel is poor,^17^ and irregular enteroid budding can prevent uniform and direct contact between cells and viral particles across the well, this protocol dissolves Matrigel and prepares a single-cell suspension, allowing for direct particle contact with cells and enriching for undifferentiated cells most likely to adhere to the Matrigel coating. In addition, our laboratory has elected to utilize a Matrigel sandwich rather than domes due to heterogeneous growth and differentiation of enteroids in the latter, a consequence of methodologically driven spatiotemporal concentration gradients of, particularly, Wnt3a.^22^ Once plated, enteroid-derived single cells are subjected to a range of puromycin concentrations to establish the minimum concentration required for selection of transduced cells.

**Figure 1:**
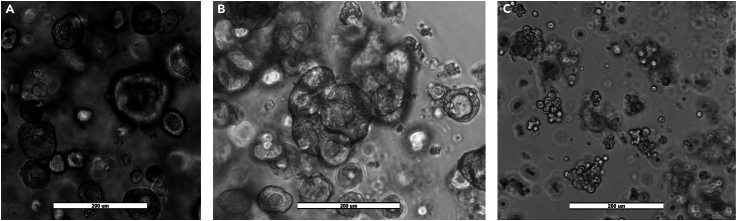
Representative bright-field photomicrographs of human preterm infant ileal enteroids Scale bars represent 200 μm. (A) Enteroids characterized largely by spheroid (stem-like) morphology, optimal for lentiviral transfection and puromycin selection. (B) Highly differentiated enteroids (budding in center of image), unlikely to result in sufficient transduction efficiency. (C) Enteroid single-cell suspension, several hours post-plating. Note single-cell morphology, but clumps of individual cells forming.

***Note:*** Enteroids characterized by spheroid morphology (Figure 1A) are optimal for both puromycin selection cytotoxicity trials and lentiviral transfection. Highly budded (differentiated) enteroids (Figure 1B) proliferate and propagate at reduced rates following transfection. In our hands, optimal morphology occurs approximately 2 days post-passaging, resulting in enteroids that are no larger than 100–150 μm in diameter.

***Note:*** Allow Matrigel to thaw on ice at 4°C for at least 12 h. Cool phosphate-buffered saline (PBS) and DMEM/F-12 (Dulbecco’s Modified Eagle Medium F12) at 4°C for at least 12 h.

***Note:*** All steps involving viable cells should take place within a sterile biological safety cabinet (here, we use a Type A2, but any appropriately rated and sterile cabinet will work).

1. Microplate Matrigel coating (Troubleshooting).
  a. Place a CoolSink XT 96F on ice a minimum of 30 min before plating.
  b. Once ice-cold, place a sterile 96-well microplate on the CoolSink XT 96F.
  c. Using a 200 μL pipette and pipette tips, add 100 μL ice-cold PBS to each experimental well. Allow PBS to sit in wells for 1 min.
  d. After 1 min, remove PBS from each well and replace with 30 μL ice-cold Matrigel.
  e. Place plate in 37°C incubator for a minimum of 20 min to solidify Matrigel. Retain plate in incubator until ready to plate cells.

***Alternative:*** We use a Heracell VIOS CO_2_ incubator, but any sterile incubator containing a humidity reservoir, high-efficiency particulate air (HEPA) filtration, heat chamber, and CO_2_ capabilities will work.

2. Passage enteroids through dissociation into single cells (Troubleshooting).
  ***Note:*** TrypLE Express is used in lieu of trypsin. TrypLE is formulated with high purity, eradicating extraneous enzymes often present in trypsin, eliminating the need for fetal bovine serum (FBS) inactivation of the enzyme, and reducing cell damage during passaging.
  ***Note:*** TrypLE Express should be warmed to 37°C in a water or bead bath prior to use.
  a. Aspirate WRNE media (containing Wnt3a, R-spondin 3, and noggin for enteroid growth^23^) from wells of a preexisting 24-well plate containing enteroids.
    ***Note:*** The number of wells of a 24-well plate required to seed a 96-well plate will depend upon enteroid line growth rates and characteristics of source tissue. The following steps are calibrated for use of 6 wells of a 24-well plate, equating to 1 × 10^5–6^ cells/well with the IL1004 enteroid line.
  b. Using a 1 mL pipette and pipette tips, add 500 μL of prewarmed TrypLE Express to each well.
  c. Mechanically disrupt Matrigel domes by pipetting up and down 5 times.
  d. Place plate in 37°C incubator for 10 min. Further mechanical disruption may be required after 10 min to break up remaining cell clusters.
    **CRITICAL:** To prevent cell death, do not leave enteroids in TrypLE Express longer than 10 min.
  e. Following the 10 min incubation, add 500 μL ice-cold DMEM/F-12 to each well to dilute TrypLE Express.
  f. Once enteroids are dissociated to single cells, place contents of wells into a 15 mL centrifuge tube.
  g. Centrifuge at 400 × *g* at 4°C for 5 min to pellet cells.
    ***Note:*** We use an Eppendorf multipurpose swing bucket rotor centrifuge with adapters fit to multiple tube sizes, but any centrifuge with appropriate capabilities will suffice.
    ***Note:*** Cells (white) will collect at the bottom of the 15 mL centrifuge tube. Remaining Matrigel will form an opaque layer immediately above the cell pellet. At 4°C, Matrigel liquifies and should be removable through aspiration or careful pipetting.
  h. Remove supernatant containing Matrigel, resuspend pellet in 4 mL DMEM/F-12, and centrifuge again at 400 × *g* and 4°C for 5 min.
  i. Remove all but 500 μL of supernatant.
    ***Note:*** If Matrigel is still visible around the cell pellet, repeat ice-cold DMEM/F-12 washes until Matrigel completely dissolves and is removed.
3. Count cells for consistent and even plating (Troubleshooting).
  ***Alternative***: We utilize the automated Countess 3 cell counter, but any automated cell counter system or manual hemocytometer will suffice.
  a. Resuspend pellet in remaining 500 μL of DMEM/F-12.
  b. Using a 20 μL pipette and pipette tips, add 15 μL of cell suspension into a 1.50 mL microcentrifuge tube.
  c. Add 15 μL 0.40% trypan blue stain and mix thoroughly via pipette.
  d. Pipette 10 μL of stained cell suspension into each of the two chambers of a Countess cell counting chamber slide.
  e. Insert one end of the chamber slide into the Countess 3, and record number and viability of cells.
  f. Repeat measurements with second slide chamber.
    ***Note:*** Our laboratory has standardized cell concentrations for a 96-well plate to 6 × 10^4^ cells/mL, but optimal plating concentrations may vary.
4. Plate enteroid single-cell suspension in Matrigel sandwich.
  a. On day 0, calculate the total volume of WRNE media required to resuspend cells such that 100 μL of final cell suspension will seed each well of the 96-well plate with an empirically determined cell density (see above Note).
    ***Note:*** Cell pelleting (as in Preparation Step 2g) may be required if tube media already exceeds target volume.
  b. Remove 96-well microplate from incubator.
  c. Pipette 100 μL of single-cell suspension onto Matrigel layer within each experimental well.
  d. Return plate to incubator for 24 h.
  e. The following day (day 1), gently aspirate media from each well, leaving only cells attached to the bottom layer of Matrigel.
  f. To finalize the Matrigel sandwich, overlay 30 μL of Matrigel on attached cells.
  g. Place microplate in a 37°C incubator for 20 min.
  h. Following 20 min incubation, add 100 μL of WRNE media (20°C–22°C) to each experimental well.
    ***Note:*** Media should be dispensed into wells against the sidewalls, slowly and gently, so as not to disturb the Matrigel sandwich.
5. Puromycin selection.
  a. The next morning (day 2), replace culture media with WRNE containing puromycin at 12 different concentrations (our laboratory uses 0–11 μg/mL), with the total volume/well (WRNE + puromycin) at 110 μL.
    ***Note:*** Allocate sufficient wells to each treatment so as to be powered to make definitive conclusions at trial end.
    ***Note:*** Cells should be visualized daily via bright-field microscopy for general morphology to aid in determining puromycin dosing duration.
    ***Alternative****:* Our laboratory uses a Revolution Hybrid automated microscope, but any microscope with 10X or 20X bright-field capabilities and microplate adaptors will be sufficient.
  b. In 2 days (day 4), replace culture media with fresh WRNE + puromycin.
  c. Repeat Preparation Step 5b, with the last WRNE + puromycin change occurring at day 6.
  d. In 3 days (day 9), determine cell viability through cell counting or commercially available viability assays.
  e. The minimum concentration of puromycin resulting in complete cell death after 5–9 days of selection represents the optimal concentration for this cell type during transfection.
    ***Note:*** This concentration may also be effective over a shortened dosing period, but dosing duration should be empirically determined by each laboratory. This optimal puromycin concentration will be used to select cells for downstream applications (e.g., western blotting, Steps 6–16).

### Lentiviral transfection calculations

**Timing: 30 min**

This step describes calculations required for determining lentiviral titer, multiplicity of infection (MOI), and transducing units. Because the relationship between reported lentiviral titer (determined by particle vendor through p24 enzyme-linked immunosorbent assay [ELISA]) and functional titer is dependent upon the viral vector and cell type to be transfected, functional titer should be optimized by each laboratory. Particularly difficult cell lines may require an MOI up to 50–100. Both the flow cytometry and western blot protocols described below are suitable in determining optimal MOI/functional titer per vector and cell line. Optimization in our laboratory for enteroid transfection has resulted in an MOI of 20.

***Alternatives:*** Techniques such as colony forming unit (CFU) assays, fluorescence-activated cell sorting (FACS) titration, or quantitative real-time reverse-transcription polymerase chain reaction (qRT-PCR) are additional commonly used techniques for optimizing functional titers.

***Note:*** Transduction efficiency can be optimized using the MISSION pLKO.1-puro-CMV-tGFP positive control transduction particles before progressing to use of targeted short hairpin ribonucleic acid (shRNA) constructs. This is a critical step in differentiating effects of a particular gene knockdown from those of transduction efficiency, should protocol troubleshooting be required.

***Note:*** In addition to viral particles targeted for knockdown of the gene of interest, appropriate control particles should also be run with each experiment. Here, we utilize both negative (MISSION pLKO.1-puro non-target shRNA control transduction particles) and positive (MISSION pLKO.1-puro-CMV-tGFP positive control transduction particles) controls.

6. Calculate lentiviral particle requirements based on desired MOI.
  a. Total transducing units (TU) = Number of lentiviral particles/well x MOI.
  b. Number of lentiviral particles/well = Total TU/lentiviral titer.

***Note:*** Lentiviral titer, expressed in TU/mL, is provided on the certificate of analysis (CoA) for many custom and off-the-shelf lentivirus particle preparations, including the MISSION viral particles used in this protocol (1 × 10^7^ TU/mL).

## Key resources table

**Table:** 

REAGENT or RESOURCE	SOURCE	IDENTIFIER
**Antibodies**		

Mouse monoclonal anti-β-actin (AC-74) antibody dilution: 1/10,000	MilliporeSigma	Cat#A2228; RRID: AB_476697
Mouse monoclonal anti-S6 ribosomal protein (54D2) antibody dilution: 1/500	Cell Signaling Technology	Cat#2317; RRID: AB_2238583
Mouse monoclonal anti-TSC2 (clone 614204) antibody dilution: 1/1,000	R&D Systems	Cat#MAB40401; RRID: AB_10719268
Peroxidase AffiniPure goat anti-mouse IgG (H + L) antibody dilution: 1/5,000	Jackson ImmunoResearch	Cat#115-035-003; RRID: AB_10015289
Peroxidase AffiniPure goat anti-rabbit IgG (H + L) antibody dilution: 1/5,000	Jackson ImmunoResearch	Cat#111-035-003; RRID: AB_2313567
Rabbit monoclonal anti-GAPDH (D16H11) XP antibody dilution: 1/10,000	Cell Signaling Technology	Cat#5174; RRID: AB_10622025
Rabbit monoclonal anti-Ki67 antibody dilution: 1/800	Abcam	Cat#ab15580; RRID: AB_443209
Rabbit monoclonal anti-p44/42 MAPK (Erk1/2) (137F5) antibody dilution: 1/1,000	Cell Signaling Technology	Cat#4695; RRID: AB_390779
Rabbit monoclonal anti-phospho-p44/42 MAPK (Erk1/2) (Thr202/Tyr204) (D13.14.4E) XP antibody dilution: 1/1,000	Cell Signaling Technology	Cat#4370; RRID: AB_2315112
Rabbit monoclonal anti-phospho-S6 ribosomal protein (Ser240/244) (D68F8) XP antibody dilution: 1/1,000	Cell Signaling Technology	Cat#5364; RRID: AB_10694233

**Chemicals, peptides, and recombinant proteins**		

2-mercaptoethanol	Thermo Fisher Scientific	Cat#21985023
4X Laemmli sample buffer	Bio-Rad	Cat#1610747
10% NBF	Fisher Scientific	Cat#22-110-873
10X Tris/glycine/SDS buffer	Bio-Rad	Cat#1610732
A 83-01	Tocris Bioscience	Cat#2939
Advanced DMEM/F-12 [-] L-glutamine	Thermo Fisher Scientific	Cat#12634028
B-27 supplement (50X), serum-free	Thermo Fisher Scientific	Cat#17504044
BSA, fraction V, fatty acid-free	MilliporeSigma	Cat#10775835001
DMEM/F-12 with 15 mM HEPES	STEMCELL Technologies	Cat#36254
DMSO	Fisher Scientific	Cat#AAJ66650AD
EGF from rat	MilliporeSigma	Cat#SRP3238
GlutaMAX supplement	Thermo Fisher Scientific	Cat#35050061
HEPES (1 M)	Thermo Fisher Scientific	Cat#15630-056
Human (Leu15)-Gastrin I	MilliporeSigma	Cat#G9145
Instant non-fat dry milk	Nestle Carnation	Cat#B082LYGPJ9
Matrigel GFR basement membrane matrix, phenol red- free, LDEV-free	Corning	Cat#356231
Methanol	MilliporeSigma	Cat#646377
*N*-2 supplement (100X)	Thermo Fisher Scientific	Cat#17502048
NAC	MilliporeSigma	Cat#A9165
Nicotinamide	MilliporeSigma	Cat#N0636
PBS, 1X [-] calcium, magnesium, phenol red	Thermo Fisher Scientific	Cat#10010023
Phosphatase inhibitor cocktail set II	MilliporeSigma	Cat#524625
Pierce HRP	Thermo Fisher Scientific	Cat#31490
PMSF	MilliporeSigma	Cat#93482
Ponceau S staining solution	Thermo Fisher Scientific	Cat#A40000279
Protease inhibitor cocktail set III, EDTA-Free	MilliporeSigma	Cat#539134
Puromycin	InvivoGen	Cat#ant-pr-1
RIPA lysis and extraction buffer	Thermo Fisher Scientific	Cat#89900
SB 202190	Tocris Bioscience	Cat#1264
SuperSignal West Pico PLUS chemiluminescent substrate	Thermo Fisher Scientific	Cat#34580
TBS, 10X, pH 7.4	Thermo Fisher Scientific	Cat#J60764.K2
TrypLE Express enzyme (1x), no phenol red	Thermo Fisher Scientific	Cat#12604013
Tween 20	MilliporeSigma	Cat#P7949
Y-27632 dihydrochloride	MilliporeSigma	Cat#Y0503

**Critical commercial assays**		

BrightComp eBeads Compensation Bead Kit	Thermo Fisher Scientific	Cat#A10514
LIVE/DEAD Fixable Red Dead Cell Stain Kit, for 488 nm excitation	Thermo Fisher Scientific	Cat#L34971
Pierce BCA Protein Assay Kit	Thermo Fisher Scientific	Cat#23225
TransDux MAX lentivirus transduction reagent	System Biosciences	Cat#LV860A-1

**Experimental models: Cell lines**		

Human preterm infant ileal enteroids (passages: 20–22)	This paper (Laboratory of Dr. Sasirekha Ramani)	IL1004
L-WRN cells containing Wnt-3a, R-spondin 3, and noggin	ATCC	Cat#CRL-3276

**Recombinant DNA**		

MISSION pLKO.1-puro-CMV-tGFP positive control transduction particles	MilliporeSigma	Cat#SHC003V
MISSION pLKO.1-puro non-target shRNA control transduction particles	MilliporeSigma	Cat#SHC016V
MISSION transduction particles (*TSC2*; TRCN0000010454); sequence: CAGCATTAATCTCTTACCATA	MilliporeSigma	Cat#SHCLNV

**Software and algorithms**		

Accuri C6 Plus Flow Cytometer Software v1.0.34.1, build 2018111.34.1	BD Biosciences	Cat#661083
EndNote v20.6	Clarivate	https://endnote.com
Graphical abstract and Figure 2A	BioRender	https://www.biorender.com
Image Lab v6.1	Bio-Rad	Cat#17006130
ImageJ v1.54g	ImageJ	https://imagej.nih.gov/ij/
Prism v9.0.2	GraphPad	https://www.graphpad.com/scientific-software/prism/
Revolution software v1.0.26.2	ECHO	https://discover-echo.com/revolution/
SoftMax Pro software v7.1.2	Molecular Devices	https://www.moleculardevices.com/products/microplate-readers/acquisition-and-analysis-software/softmax-pro-software

**Other**		

4%–20% Mini-PROTEAN TGX precast protein gels, 10- well, 50 μL	Bio-Rad	Cat#4561094
5 mL round-bottom high-clarity polypropylene test tubes, with snap cap, sterile	Corning	Cat#352063
10 μL filter pipette tips	Neptune Scientific	Cat#BT10
96-well, Nunclon delta-treated, flat-bottom microplate, Sterile	Thermo Fisher Scientific	Cat#167425
200 μL filter pipette tips	Neptune Scientific	Cat#BT200
1,000–1,250 μL pipette tips	Neptune Scientific	Cat#BT1250
1300 Series Class II, type A2 biological safety cabinet	Thermo Fisher Scientific	Cat#1353
Accuri C6 Plus flow cytometer	BD Biosciences	Cat#661311
Adhesive plate seals	Fisher Scientific	Cat#08–408-240
Aerosol barrier pipette tips, 2–20 μL	Fisher Scientific	Cat#02–707-432
Analog vortex mixer	Fisher Scientific	Cat#02–215-414
Blot development folders	Advansta	Cat#L-07020
ChemiDoc XRS+ System	Bio-Rad	Cat#5837
Cimarec+ Stirrer Series 10.25 × 10.25 in, ceramic	Thermo Fisher Scientific	Cat#S88850100
Clear, flat-bottom, immuno, non-sterile, 96-well plate	Thermo Fisher Scientific	Cat#442404
CoolSink XT 96F, flat bottom plate module	Corning	Cat#432070
Countess 3 automated cell counter	Thermo Fisher Scientific	Cat#A49862
Countess cell counting chamber slides	Thermo Fisher Scientific	Cat#C10228
Digital dry bath/block heater	Thermo Fisher Scientific	Cat#88870002
Finnpipette F2 0.2–2 μL pipette	Thermo Fisher Scientific	Cat#4642010
Finnpipette F2 2–20 μL pipette	Thermo Fisher Scientific	Cat#4642050
Finnpipette F2 20–200 μL pipette	Thermo Fisher Scientific	Cat#4642080
Finnpipette F2 100–1000 μL pipette	Thermo Fisher Scientific	Cat#4642090
Fisherbrand gel-loading tips, 1–200 μL	Fisher Scientific	Cat#02–707-138
General purpose bath	Thermo Fisher Scientific	Cat#TSGP10
Heracell VIOS 160i incubator	Thermo Fisher Scientific	Cat#51033774
High-performance 15 mL centrifuge tubes with flat caps	VWR	Cat#89039-666
LSE platform rocker, single platform, 115V	Corning	Cat#6703
Micro 17/17R microcentrifuge	Fisher Scientific	Cat#13-100-675
Mini-PROTEAN Tetra vertical electrophoresis cell for Mini precast gels, 2-gel	Bio-Rad	Cat#1658005
Mini Trans-Blot electrophoretic transfer cell	Bio-Rad	Cat#1703930
Nunc cell culture/Petri dishes, 150 × 21 mm	Thermo Fisher Scientific	Cat#168381
Parafilm M, Bemis	VWR	Cat#52858-000
PowerPac HC power supply	Bio-Rad	Cat#1645052
Precision Plus protein dual color standards	Bio-Rad	Cat#1610374
Revolution Hybrid automated microscope	ECHO	Cat#RON-K
Snap-cap low retention 1.50 mL microcentrifuge tubes	Thermo Fisher Scientific	Cat#3448PK
SpectraMax iD3 multi-mode microplate reader	Molecular Devices	Cat#5054747
Spinbar magnetic stir bar, octagon, 41 mm	VWR	Cat#58947-114
Swing bucket rotor centrifuge	Eppendorf	Cat#5910R
Synergy UV water purification system	MilliporeSigma	Cat#SYNSVHFWW
Thermo-Flask benchtop liquid nitrogen 2 L container	Thermo Fisher Scientific	Cat#116704B
Thick blot filter paper, precut, 7.5 × 10 cm	Bio-Rad	Cat#1703932
Transfer membrane roll 300 × 3,000 mm, PVDF, 0.45 μm	GVS	Cat#1212639
Trypan blue stain, 0.40%	Thermo Fisher Scientific	Cat#T10282
Tube revolver rotator	Thermo Fisher Scientific	Cat#88881001

## Materials and equipment

### Puromycin selection cytotoxicity trial

• CoolSink XT 96F (Corning, 432070).
• TrypLE Express (Thermo Fisher Scientific, 12604013).
• Sterile 96-well microplate (Thermo Fisher Scientific, 167425).
• Matrigel, growth factor reduced (GFR; Corning, 356231).
• DMEM/F-12 with 15 mM HEPES (STEMCELL Technologies, 36254).
• Dimethyl sulfoxide (DMSO; Fisher Scientific, AAJ66650AD).
• 15 mL centrifuge tubes (VWR, 89039–666).
• Countess 3 automated cell counter (Thermo Fisher Scientific, A49862).
• Countess cell counting chamber slides (Thermo Fisher Scientific, C10228).
• Trypan blue stain, 0.40% (Thermo Fisher Scientific, T10282).
• 1.50 mL microcentrifuge tubes (Thermo Fisher Scientific, 3448PK).
• PBS (Thermo Fisher Scientific, 10010023).
• Bovine serum albumin (BSA; Millipore Sigma, 10775835001).
• L-WRN conditioned medium, generated from L-WRN cell line (ATCC, CRL-3276).
• Advanced DMEM/F-12, [-] L-glutamine (Thermo Fisher Scientific, 12634028).
• GlutaMAX supplement (Thermo Fisher Scientific, 35050061).
• 2-[4(2-hydroxyethyl)piperazin-1-yl)ethanesulfonic acid (HEPES; Thermo Fisher Scientific, 15630–056).
• N-acetyl-L-cysteine (NAC: Millipore Sigma, A9165): Dissolve 500 mg in 6.13 mL MilliQ H2O (obtained from an ultrapure water purification system) to make 500 mM stock. Store in 1 mL aliquots at −20°C.
• Epidermal growth factor from rat (EGF; Millipore Sigma, SRP3238): Prior to opening, centrifuge the vial. Reconstitute 100 μg in 3 mL MilliQ water or PBS. For long-term storage, dilute further in 0.10% BSA, aliquot to 1 mL vials, and place at −20°C or −80°C.
• A 83–01 (Tocris Bioscience, 2939): Prior to opening, centrifuge the vial. Reconstitute 1 mg in 4.75 mL DMSO to make 500 mM stock. Store in 1 mL aliquots at −20°C.
• Human Leu^15^-Gastrin I (Millipore Sigma, G9145): Reconstitute 0.10 mg in 4.81 mL PBS. Store in 1 mL aliquots at −20°C.
• Nicotinamide (Millipore Sigma, N0636): Dissolve 1.25 *g* in 10.24 mL MilliQ H_2_O. Use immediately or store up to 1 week at 4°C.
• SB 202190 (Tocris Bioscience, 1264): Dissolve 10 mg in 3.02 mL DMSO to make 10 mM stock. Store in 1 mL aliquots at −20°C.
• B-27 supplement (Thermo Fisher Scientific, 17504044): Store at −20°C until use. Thaw at 4°C. Avoid repeated freeze/thaw cycles.
• N-2 supplement (Thermo Fisher Scientific, 17502048): Store at −20°C until use. Thaw at 4°C. Avoid repeated freeze/thaw cycles.
• Y-27632 (Millipore Sigma, Y0503): Dissolve 1 mg in 624 μL PBS or double-distilled H_2_O to make 5 mM stock. Store in 200 μL aliquots at −20°C for up to 1 year.
• Puromycin (InvivoGen, ant-pr-1): From a 1 mL puromycin vial, dilute 5.44 μL puromycin into 94.56 μL antibiotic-free WRNE media to generate a 1 mM stock. Aliquot into smaller volumes to avoid freeze/thaw cycles during longer storage.
• WRNE media.

To prepare 50 mL of WRNE enteroid growth media, combine ingredients below. For preparation of large media batches, see Miyoshi and Stappenbeck^11^ or VanDussen et al.^24^ Our laboratory maintains L-WRN cells producing Wnt-3a, R-spondin 3, and noggin, but conditioned media generated from this cell line is also commercially available from large biomedical vendors.

**Table:** WRNE media

Reagent	Stock concentration	Final concentration	Amount
50% L-WRN conditioned medium	–	50%	25 mL
Advanced DMEM/F-12	–	45%	23 mL
GlutaMAX supplement	200 mM	2 mM	0.5 mL
HEPES	1 M	10 mM	0.5 mL
B-27 supplement	50X	1X	1 mL
*N*-2 supplement	100X	1X	500 μL
*N*-acetyl-L-cysteine	500 mM	1 mM	100 μL
EGF from rat	50 μg/mL	50 ng/mL	50 μL
A 83-01	500 μM	500 nM	50 μL
Human Leu^15^-Gastrin I	10 μM	10 nM	50 μL
Nicotinamide	1 M	10 mM	500 μL
SB 202190	10 mM	10 μM	50 μL
Y-27632	5 mM	10 μM	100 μL
**Total**	**N/A**	**N/A**	**50 mL**

Store at 4°C for up to 1 week or at −20°C for up to 6 months.

### Lentiviral transfection calculations

• MISSION shRNA lentiviral transduction particles (*TSC2*; TRCN0000010454; Millipore Sigma, SHCLNV).
• MISSION pLKO.1-puro-CMV-tGFP positive control transduction particles (Millipore Sigma, SHC003V).
• MISSION pLKO.1-puro non-target shRNA control transduction particles (Millipore Sigma, SHC016V).

### Transfecting enteroids

• Sterile 96-well microplates (Thermo Fisher Scientific, 167425).
• Matrigel, GFR (Corning, 356231).
• CoolSink XT 96F (Corning, 432070).
• PBS (Thermo Fisher Scientific, 10010023).
• TrypLE Express (Thermo Fisher Scientific, 12604013).
• DMEM/F-12 with 15 mM HEPES (STEMCELL Technologies, 36254).
• 15 mL centrifuge tubes (VWR, 89039–666).
• Countess 3 automated cell counter (Thermo Fisher Scientific, A49862).
• Countess cell counting chamber slides (Thermo Fisher Scientific, C10228).
• Trypan blue stain, 0.40% (Thermo Fisher Scientific, T10282).
• 1.50 mL microcentrifuge tubes (Thermo Fisher Scientific, 3448PK).
• TransDux MAX Lentivirus Transduction Reagent (System Biosciences, LV860A-1).
• WRNE media.
• MISSION shRNA lentiviral transduction particles (*TSC2*; TRCN0000010454; Millipore Sigma, SHCLNV).
• MISSION pLKO.1-puro-CMV-tGFP positive control transduction particles (Millipore Sigma, SHC003V).
• MISSION pLKO.1-puro non-target shRNA control transduction particles (Millipore Sigma, SHC016V).
• Parafilm (VWR, 52858–000).
• Puromycin (InvivoGen, ant-pr-1).
• Transduction media.

To prepare ∼100 μL of transduction media (for 1 well of a 96-well microplate), combine the following.

**Table:** Transduction media

Reagent	Final concentration	Amount
WRNE media	–	80 μL
MAX Enhancer	1X	20 μL
TransDux	1X	0.4 μL
**Total**	**N/A**	**100.4 μL**

Store at 4°C for up to 1 day.

### Flow cytometry analysis for transduction efficiency and cell viability

• LIVE/DEAD Fixable Red Dead Cell Stain Kit, for 488 nm excitation (Thermo Fisher Scientific, L34971).
• PBS (Thermo Fisher Scientific, 10010023).
• BSA (Millipore Sigma, 10775835001).
• TrypLE Express (Thermo Fisher Scientific, 12604013).
• 5 mL polypropylene tubes (Corning, 352063).
• Trypan blue stain, 0.40% (Thermo Fisher Scientific, T10282).
• 1.50 mL microcentrifuge tubes (Thermo Fisher Scientific, 3448PK).
• Countess 3 automated cell counter (Thermo Fisher Scientific, A49862).
• Countess cell counting chamber slides (Thermo Fisher Scientific, C10228).
• 10% neutral buffered formalin (NBF; Fisher Scientific, 22-110-873).
• BrightComp eBeads Compensation Bead Kit (Thermo Fisher Scientific, A10514).
Western blotting.
• CoolSink XT 96F (Corning, 432070).
• TrypLE Express (Thermo Fisher Scientific, 12604013).
• 1.50 mL microcentrifuge tubes (Thermo Fisher Scientific, 3448PK).
• PBS (Thermo Fisher Scientific, 10010023).
• Radioimmunoprecipitation assay (RIPA) lysis and extraction buffer (Thermo Fisher Scientific, 89900).
• Phenylmethylsulfonyl fluoride (PMSF; Millipore Sigma, 93482).
• Protease inhibitor cocktail set III (Millipore Sigma, 539134).
• Phosphatase inhibitor cocktail set II (Millipore Sigma, 524625).
• Liquid nitrogen dewar (Thermo Fisher Scientific, 116704B).
• Pierce Bicinchoninic (BCA) Protein Assay Kit (Thermo Fisher Scientific, 23225).
• BSA (Millipore Sigma, 10775835001).
• 15 mL centrifuge tubes (VWR, 89039–666).
• Clear, flat-bottom, 96-well plate (Thermo Fisher Scientific, 442404).
• Adhesive plate seals (Fisher Scientific, 08–408-240).
• 10X Tris/glycine/sodium dodecyl sulfate (SDS) buffer (Bio-Rad, 1610732).
• 1X SDS running buffer: To make 1 L of buffer, combine 10% 10X tris/glycine/SDS buffer (100 mL) and 90% MilliQ water (900 mL). Store at 4°C for up to 6 months. This buffer can be reused 4–6 times.
• 4X Laemmli sample buffer (Bio-Rad, 1610747).
• 2-mercaptoethanol (Thermo Fisher Scientific, 21985023).
• 4X SDS denaturing loading buffer: To make 1 mL of buffer, combine 90% 4X Laemmli sample buffer (900 μL) and 10% 2-mercaptoethanol (100 μL). Store in 100 μL aliquots at −80°C for up to 1 year.
• 4–20% Mini-PROTEAN TGX precast protein gels, 10-well, 50 μL (Bio-Rad, 4561094).
• Mini-PROTEAN Tetra vertical electrophoresis cell for Mini precast gels, 2-gel (Bio-Rad, 1658005).
• Precision Plus Protein dual color standards (Bio-Rad, 1610374).
• PowerPac HC power supply (Bio-Rad, 1645052).
• Spinbar magnetic stir bar, octagon, 41 mm (VWR, 58947–114).
• Polyvinylidene fluoride (PVDF) membranes (GVS, 1212639).
• Disposable Petri dishes (Thermo Fisher Scientific, 168381).
• Methanol (Millipore Sigma, 646377).
• Filter paper (Bio-Rad, 1703932).
• Mini Trans-Blot electrophoretic transfer cell (Bio-Rad, 1703930).
• Ponceau S staining solution (Thermo Fisher Scientific, A40000279).
• Tris-buffered saline (TBS; Thermo Fisher Scientific, J60764.K2).
• Tween 20 (Millipore Sigma, P7949).
• Instant Non-Fat Dry Milk (Nestle Carnation, B082LYGPJ9).
• Primary and secondary antibodies (see key resources table).
• Horseradish peroxidase (HRP; Thermo Fisher Scientific, 31490).
• SuperSignal West Pico PLUS chemiluminescent substrate (Thermo Fisher Scientific, 34580).
• Blot development folders (Advansta, L-07020).
• ImageJ (Fiji, v1.54g).
• Western lysis buffer.

To prepare 500 μL of western lysis buffer, combine the following.

**Table:** Western lysis buffer

Reagent	Final concentration	Amount
RIPA lysis and extraction buffer	97%	485 μL
PMSF	1 mM	5 μL
Protease inhibitor cocktail set III	1%	5 μL
Phosphatase inhibitor cocktail set II	1%	5 μL
**Total**	**N/A**	**500 μL**

Store at 4°C for up to 4 h.

• Western transfer buffer.

To prepare 1 L of western transfer buffer (pH 8.3), combine the following.

**Table:** Western transfer buffer

Reagent	Final concentration	Amount
10X Tris/glycine/SDS	1X	100 mL
Methanol	10%	100 mL
MilliQ H2O	80%	800 mL
**Total**	**N/A**	**1 L**

Store at 4°C for up to 1 year. This buffer can be reused 3–4 times.

• TBS, 0.10% Tween 20 (TBST).

To prepare 1 L of TBST, combine the following.

**Table:** TBST

Reagent	Final concentration	Amount
TBS, 10X	1X	100 mL
Tween 20	0.10% (v/v)	1 mL
MilliQ H2O	89.9%	899 mL
**Total**	**N/A**	**1 L**

Store at 20°C–22°C for up to 4 months.

• TBST with 3% BSA.

To prepare 1 L of TBST with 3% BSA, combine the following.

**Table:** TBST with 3% BSA

Reagent	Final concentration	Amount
BSA	3% (w/v)	1.50 *g*
TBS, 10X	1X	100 mL
Tween 20	0.10% (v/v)	1 mL
MilliQ H2O	–	Adjust to 1000 mL
**Total**	**N/A**	**1 L**

Store at 4°C for up to 2 weeks.

• TBST with 5% non-fat milk.

To prepare 1 L of TBST with 5% non-fat milk, combine the following.

**Table:** TBST with 5% non-fat milk

Reagent	Final concentration	Amount
Milk	5% (w/v)	2.50 *g*
TBS, 10X	1X	100 mL
Tween 20	0.10% (v/v)	1 mL
MilliQ H2O	–	Adjust to 1000 mL
**Total**	**N/A**	**1 L**

Store at 4°C for up to 2 weeks.

## Step-by-step method details

### Transfecting enteroids

**Timing: 3 h**

This step dissociates enteroids into a single-cell suspension. Cells are then plated in a 96-well plate between two layers of Matrigel (Matrigel sandwich) for consistent access to nutrients. Transduction media contains TransDux MAX, a transduction solution similar to polybrene but with increased efficacy in primary cells. Toxicity of TransDux MAX, or similar transduction solutions, should be evaluated in cells of interest before attempting transfection, as some primary cell lines, in particular, are sensitive to these agents.

***Note:*** Lentiviral particles are vulnerable to significant temperature shifts. Avoid freeze/thaw cycles and exposure to ambient temperatures by retaining particles on ice during use. Aliquot remaining particles into smaller working volumes and freeze at −80°C. Aliquots of lentiviral particles should always be thawed on ice.

***Note:*** When working with lentiviruses, care should be taken to avoid surface contamination. Decontaminate equipment and disposable supplies, both liquid and solid, with 20 min of 10% bleach or a similar oxidizing or decontaminating agent dictated via institutional/country regulations.

***Note:*** Place 2 96-well, flat-bottom sterile microplates in a 37°C, 5% CO2 incubator for at least 12 h.

1. Transfect enteroid-derived cells (Troubleshooting).
  a. Via simple bright-field microscopy, ensure quality (more spheroids, reduced budding) and quantity of enteroids before initiating transfection (Figure 1).
  b. Coat one 96-well microplate with Matrigel, as per Preparation Step 1.
  c. Dissociate enteroids into a single-cell suspension, as per Preparation Step 2.
  d. Count cells, as per Preparation Step 3.
  e. Centrifuge tubes at 400 × *g* at 4°C for 5 min to pellet cells.
  f. Remove supernatant. Calculate the total volume of transduction media required to resuspend cells such that 100 μL of final cell suspension will seed each well of the 96-well plate with an empirically determined cell density (for our laboratory, 6 × 10^4^ cells/mL, or a cell number sufficient to coat approximately 75% of each well).
    ***Note:*** Cell pelleting (as in Step 2g) may be required if tube media already exceeds target volume.
  g. Add MISSION lentiviral transduction particles at an MOI of 20 (or alternative, as optimized in Preparation Step 6) to transfect cells.
  h. Plate 100 μL/well of cell suspension in each well of the remaining, uncoated 96-well plate.
    ***Note:*** Refer to Preparation Step 6 to calculate number of lentiviral particles needed per well.
  i. Seal microplate with parafilm to prevent viral contamination.
  j. Spinoculate plate via centrifugation at 600 × *g* and 32°C for 1 h.
  k. Following spinoculation, verify with bright-field microscopy that cells are localized to well bottoms.
  l. Add 100 μL 20°C–22°C WRNE to each well, remaining careful not to disturb cells localized to bottom of wells.
  m. Pipette up and down to detach cells from well bottoms and transfer contents of 3 wells to a 1.50 mL microcentrifuge tube.
  n. Centrifuge tubes at 600 × *g* at 4°C for 5 min.
    ***Note:*** Our laboratory uses contents of 3 wells in a 1.50 mL microcentrifuge tube because this tube size is convenient for the refrigerated microcentrifuge we utilize (see below, Alternative). However, additional tube volumes/well numbers can be utilized, as convenient.
    ***Alternative****:* Our laboratory uses a Micro 17/17R refrigerated microcentrifuge from Fisher. Centrifuge speeds depend upon the rotor radius, but any microcentrifuge with appropriate speed settings, cooling capabilities, and tube holders will suffice.
  o. Discard supernatant and resuspend cell pellet in 300 μL WRNE.
  p. Pipette 100 μL of cell suspension into each experimental well of the Matrigel-coated 96-well microplate, and place microplate in 37°C incubator for 24 h.
  q. The following day, aspirate media from each well, leaving only live, attached cells on bottom layer of Matrigel sandwich.
  r. Overlay 30 μL of Matrigel onto cells, completing the Matrigel sandwich.
  s. Incubate plate at 37°C and 5% CO_2_ for 20 min.
  t. Following incubation, pipette 100 μL of WRNE onto each Matrigel sandwich.
    **CRITICAL:** Add 100 μL PBS to unused wells to avoid evaporation in experimental wells.
    ***Note:*** Transfected cells should express green fluorescent protein (GFP) within 36–48 h post- transfection.
  u. Replace media with fresh WRNE every other day.
  v. A minimum of 4 days post-transfection, collect cells for flow cytometry measurement of transduction efficiency (Steps 2–5).
    ***Note:*** If utilizing cells for downstream analyses (*e.g.*, western blot), first select for transduced cells utilizing puromycin at the empirically determined concentration and dosing period resulting from Preparation Step 5e.

### Flow cytometry analysis for transduction efficiency and cell viability

**Timing: 6 h**

This step outlines the use of flow cytometry as a method for determining transduction efficiency and cell viability effects on enteroids. MISSION lentiviral particles are tagged with tGFP. Utilization of a vector incorporating tGFP allows for easy identification of transfected versus uninfected cells. In addition, the effects of lentiviral transfection on viability can be assessed through use of a fluorescent reactive dye. In dead cells, the dye penetrates compromised membranes, reacting with free amines both intracellularly and on the cell surface, producing a vibrant fluorescent staining pattern. In live cells, the dye interacts exclusively with cell surface amines, generating a reduced signal and allowing for discrimination between live and dead cells. This dye is functional on both live cells and those that have been fixed following dye administration.

2. Prepare viability dye and cell diluent.
  a. From the LIVE/DEAD Fixable Red Dead Cell Stain Kit, allow component A (red fluorescent reactive dye) and component B (anhydrous DMSO), stored at −80°C, to reach 20°C–22°C.
    ***Note:*** Ensure DMSO is thawed before opening vials.
  b. Add 50 μL DMSO to vial of red reactive dye. Thoroughly mix contents and visually confirm complete dissolution of dye.
    ***Note:*** Dye should be used within a few hours of preparation. Reconstituted dye can be aliquoted into smaller volumes and stored at −80°C for a maximum of 3 months.
  c. Prepare PBS containing 1% BSA (for 10 mL, add 0.10 *g* BSA). Place solution on ice.
3. Dissociate enteroids into single-cell suspension.
  a. Warm TrypLE Express in a 37°C water or bead bath.
  b. Aspirate media from each well of a 96-well plate of transfected enteroids.
  c. Add 200 μL prewarmed TrypLE Express to each well.
  d. Mechanically disrupt Matrigel sandwiches in each well 10 times and place plate in 37°C incubator for 10 min.
  e. Following incubation, add 100 μL ice-cold PBS to dilute TrypLE.
  f. Pipette up and down 10–20 times to further dissociate enteroids.
  g. Transfer contents of one group of wells (defined by treatment group) into 5 mL polypropylene round-bottom tube.
  h. Repeat Step 3g for remaining groups.
  i. Centrifuge 5 mL polypropylene tubes at 400 × *g* and 4°C for 5 min to pellet cells.
  j. Remove supernatant and replace with 1 mL PBS to wash cells.
  k. Repeat Step 3i.
  l. Remove supernatant and resuspend cells in 500 μL PBS.
  m. Count cells (as per Preparation Steps 3b-f) using either the Countess 3 or a standard hemocytometer.

**CRITICAL:** A minimum of 1 × 10^5^ cells per condition is needed to perform flow cytometry.

**Figure 2:**
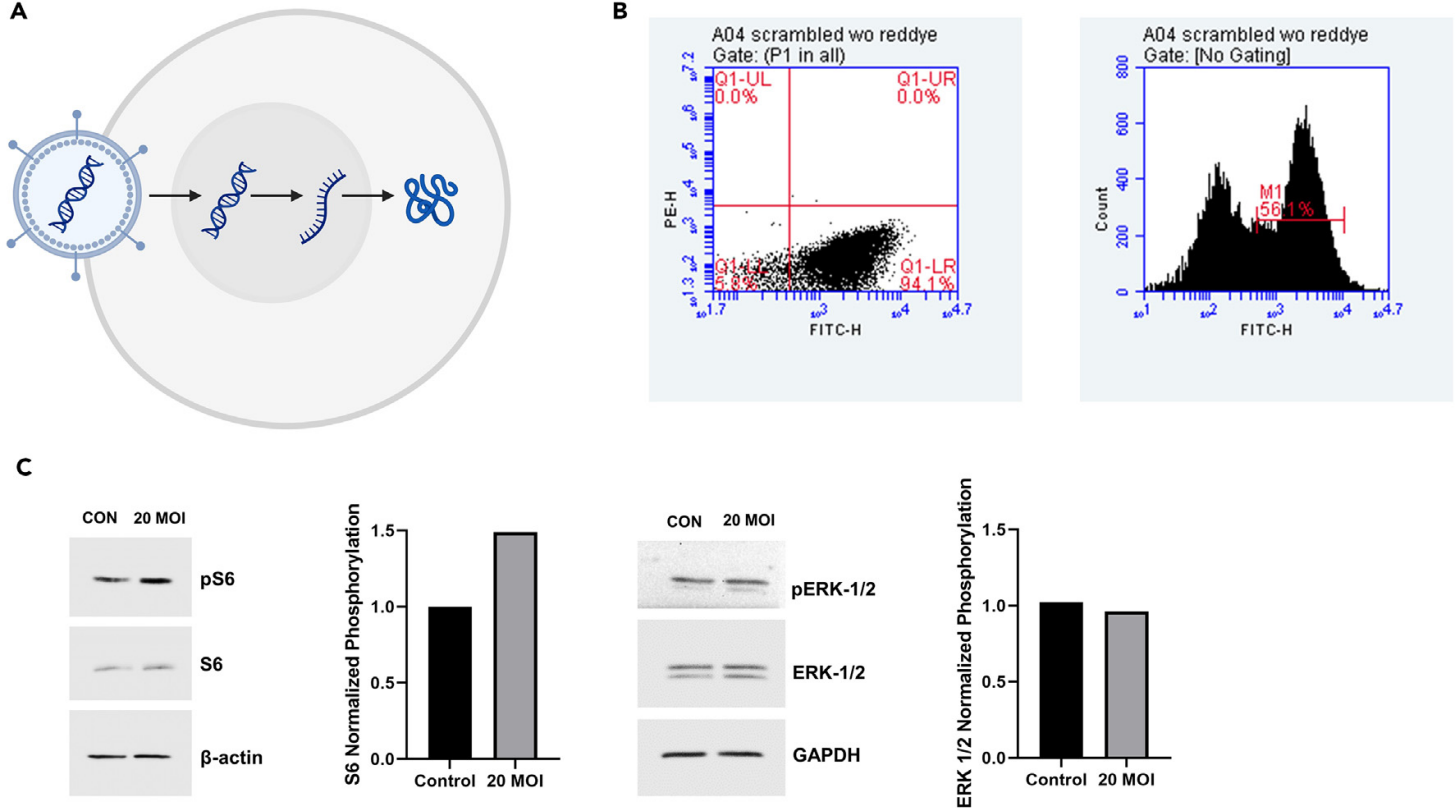
Downstream analyses of transduction efficiency and gene knockdown effects (A) Schematic of lentiviral transfection of mammalian cell. (B) Representative flow cytometry scatterplot (left, 4 quadrants), GFP expression in non-target shRNA transfection: Q1-UL = dead cells not expressing GFP; Q1-UR = dead cells expressing GFP; Q1-LL = live cells, not expressing GFP; Q1-LR = live cells, expressing GFP. On right, a histogram depicting FITC intensity along X-axis and number of events at that intensity on the Y-axis. (C) Representative western blot images for control (non-target shRNA transfection) and *TSC2* knockdown (20 MOI) in preterm human enteroids. S6, pS6, extracellular signal-regulated kinase 1/2 (ERK-1/2), and p-ERK- 1/2 are shown normalized to β-actin and glyceraldehyde 3-phosphate dehydrogenase (GAPDH), respectively. Normalized phosphorylation S6 and ERK-1/2 is quantified in bar graphs. Enteroid *TSC2* knockdown indicates an increase in phosphorylation of the ribosomal S6 protein, but no change was observed in the phosphorylation of ERK-1/2, an upstream target of mTOR.
  k. Plots are exported. Raw data are also exported as .fsc files for additional analysis.

4. Cell viability staining (Troubleshooting).
  a. Centrifuge 5 mL polypropylene tubes at 400 × *g* for 5 min at 4°C.
  b. Discard supernatant and resuspend cell pellet in 1 mL ice-cold PBS.
    ***Note:*** When using amino-reactive dye for staining, avoid resuspension/washing of cells in buffers containing extraneous proteins, such as BSA.
  c. Using a 2 μL pipette and 10 μL tips, add 1 μL reconstituted (Steps 2a-b) red reactive fluorescent dye to each 5 mL tube. Pipette up and down to mix well.
  d. Incubate tubes at 20°C–22°C for 30 min in the dark.
  e. Following 30 min incubation, centrifuge 5 mL tubes at 400 × *g* and 4°C for 5 min to pellet cells.
  f. Remove supernatant and wash cells with 1 mL PBS.
  g. Repeat Step 4e.
  h. Discard supernatant and resuspend cell pellet in 1 mL 10% NBF.
  i. Incubate cell suspension at 20°C–22°C for 15 min in the dark.
  j. Centrifuge 5 mL tubes at 400 × *g* and 4°C for 5 min to pellet cells.
  k. Discard supernatant and wash cells with 1 mL ice-cold PBS containing 1% BSA.
  l. Repeat Steps 4j-k.
  m. Place 5 mL tubes on ice.
    **Pause point:** Fixed, stained cells can be kept in the dark at 4°C for a maximum of 2 h.
5. Flow cytometry analysis (Troubleshooting steps 6 and 7).
  ***Alternative***: Our laboratory uses a BD Biosciences Accuri C6 Plus flow cytometer equipped with 488 and 640 nm lasers, but any flow cytometer with the appropriate lasers and detectors will work for this purpose.
  ***Note:*** Pipette up and down several times before infusing sample into cytometer. Users may also wish to filter cell suspension with a 37 μm cell strainer to eliminate clumping immediately prior to analysis.
  a. After performing quality control per manufacturer instructions, open the Accuri C6 Plus Flow Cytometer Software, which automatically directs user to the Collect tab.
  b. Manually calibrate the flow cytometer by establishing compensation settings with GFP compensation beads.
    ***Alternative***: We use the BrightComp eBeads GFP Compensation Bead Kit, but stably transfected GFP- expressing cell lines can also be used for compensation.
  c. Select an empty sample well and enter a sample name in the text box above the 96-well grid.
  d. Set acquisition parameters to 10,000 events, slow (14 μL/min) fluidics rate, and a forward scatter (FSC) threshold between 200,000 and 500,000. A secondary threshold is set at 640 nm to eliminate background autofluorescence in the red fluorescence channel.
  e. GFP (fluorescein isothiocyanate, FITC) is collected in the FL1 channel (530/30) and red (phycoerythrin, PE; viability) is collected in the FL3 channel (> 670). Both are plotted logarithmically.
  f. Manually load the first sample over the sample injection port.
  g. Click run to start the sample collection and save the workspace when prompted.
  h. FSC vs. side scatter (SSC) dot plots are displayed by default under the Analyze tab, and gating is applied to identify single cells.
  i. Additional plots can be generated via altering X- and Y-axes to desired filter channels. Gating from initial plots is automatically applied to subsequent plots.
  j. Gating is applied to identify live and transfected (Figure 2B) cells using PE and FITC filter channels, respectively.

### Western blotting for proteins downstream of *TSC2*

**Timing: 4 days**

This step describes the use of western blotting for downstream evaluation of lentiviral gene knockdown on biological processes.

***Note:*** Place PBS at 4°C for at least 12 h before enteroid harvest.

6. Enteroid collection.
  a. Place a CoolSink XT 96F on ice a minimum of 30 min before enteroid harvest.
  b. Remove 96-well microplate containing enteroids from incubator.
  c. Place microplate on pre-cooled CoolSink XT 96F, and maintain on ice.
  d. Aspirate WRNE media from each experimental well of the 96-well microplate.
  e. Add 200 μL TrypLE Express to each well, and keep microplate on ice for 1 min.
  f. Mechanically disrupt Matrigel sandwiches by pipetting up and down 5–10 times, being careful not to introduce bubbles.
  g. Collect contents of 3 wells in a 1.50 mL microcentrifuge tube.
  h. Repeat Step 6g as many times as required to collect contents from all experimental wells.
  i. Place microcentrifuge tubes on a tube revolver rotator set to 33 revolutions per minute (rpm) at 20°C–22°C for 15 min.
    ***Alternative***: Our laboratory uses a tube revolver rotator from Thermo Fisher Scientific, but any rotator with the appropriate speed settings and tube adaptors will work.
  j. After 15 min rotation, place tubes on ice for 1 h, occasionally inverting, to dissolve Matrigel.
  k. After 1 h of cooling, centrifuge 1.50 mL microcentrifuge tubes at 600 × *g* for 5 min at 4°C.
  l. Aspirate supernatant and wash with 500 μL ice-cold PBS.
  m. Repeat Steps 6k-l until all Matrigel has been removed.
  n. Centrifuge tubes at 600 × *g* for 5 min at 4°C.
  o. Remove supernatant and replace with 300 μL ice-cold western lysis buffer.
  p. Gently pipette up and down to resuspend cells in buffer.
  q. Place 1.50 mL microcentrifuge tubes on tube revolver rotator at 20 rpm for 15 min at 4°C.
  r. Centrifuge microcentrifuge tubes at 14,000 × *g* for 15 min at 4°C to pellet cell debris.
  s. Transfer supernatant containing cell protein to a new 1.50 mL microcentrifuge tube and immediately flash freeze in liquid N_2_.
    **Pause point:** Cell lysate can be stored at −80°C for up to 3 months before western blot analysis.
7. Protein quantification via BCA assay.
  a. Sample protein concentrations are quantified using the Pierce BCA Protein Assay Kit (microplate procedure, sample to working reagent [WR] ratio = 1:8), per manufacturer’s instructions.
  b. Prepare diluted BSA standards (0–2000 μg/mL) in 15 mL centrifuge tubes, using western lysis buffer as diluent.
  c. Calculate required WR volume as: (# of standards or unknowns) x (3 replicates) x (200 μL).
  d. Prepare WR in a 15 mL centrifuge tube via combining BCA Reagents A and B in a 50:1 ratio.
  e. Pipette 25 μL of standard or sample into each well of a 96-well, clear, flat-bottom, microplate.
  f. Add 200 μL of WR to each well.
  g. Mix contents of wells thoroughly for 30 s using the plate reader shaking function.
  h. Cover plate with adhesive plate cover and place in a 37°C incubator for 30 min.
  i. Cool plate to 20°C–22°C.
  j. Measure absorbance on a plate reader at 562 nm.
    ***Alternative***: Our laboratory uses a SpectraMax iD3 multi-mode microplate reader, but any plate reader with the appropriate plate holders and spectral capabilities will suffice.
  k. Subtract the average blank 562 nm absorbance from that of all other standards and samples.
  l. Prepare a standard curve by plotting the average blank-corrected absorbance for each BSA standard vs. associated μg/mL concentration.
  m. Use the standard curve to calculate protein concentration of each sample.
8. Sample preparation for electrophoresis.
  a. In 1.50 mL microcentrifuge tubes, adjust sample protein concentration to 20–25 μg/mL using 4X SDS denaturing loading buffer as a diluent.
    ***Note:*** Our samples usually result in a protein concentration averaging 1 mg/mL, which we then dilute to 20–25 μg/mL with SDS denaturing loading buffer.
  b. Place 1.50 mL microcentrifuge tubes in a 95°C heat block for 7–10 min.
    ***Alternative***: Our laboratory utilizes a digital dry bath/block heater from Thermo Fisher Scientific, but any similar instrument will work.
  c. After 7–10 min heating, place samples on ice for 10 min to ensure complete denaturation of proteins.
  d. Following cooling, vortex samples briefly.
    ***Alternative***: Our laboratory uses an analog vortex mixer from Fisher Scientific, but a vortexer from any vendor with similar capabilities will work.
  e. Centrifuge samples at 13,500 × *g* and 22°C for 2 min.
9. Electrophoresis.
  a. Remove the 4–20% Mini-PROTEAN TGX precast protein gel from storage pouch.
  b. Remove comb and green tape, and rinse wells thoroughly with 1X SDS running buffer.
  c. Repeat for second gel, if applicable.
  d. Using components of the Mini-PROTEAN Tetra Cell, 2-gel system, set the electrode assembly to the open position on a clean, flat surface.
  e. Place the first gel cassette (short plate facing inward, gel resting at a 30° angle away from center) onto gel supports.
  f. Similarly, place second gel on opposite side of clamping frame.
  g. Lock gels into place and create a leak-proof seal by gently pushing both gels toward each other. The edge of the short plates should sit just below the notch at the top of the green gasket.
  h. Gently squeeze the gel cassettes against the green gaskets. Slide green arms of the clamping frame over the gels, one at a time.
  i. Place the electrophoresis module into the tank.
  j. Fill the inner (200 mL) and outer (550 mL) buffer chambers with 1X SDS running buffer.
  k. Place a stir bar in outer buffer chamber to increase buffer infiltration of electrode assembly.
  l. Using 200 μL gel-loading tips, load 10 μL of the dual color protein ladder into the first gel lane. In each of the remaining 9 gel lanes, load 50 μL of prepared sample.
  m. Place lid on the Mini-PROTEAN Tetra tank, making sure to align color-coded plugs and jacks.
  n. Insert electrical leads into power supply.
  o. Run gels at a power output of 80 V on a magnetic mixer with continuous stirring.
    ***Alternative***: Our laboratory uses a magnetic stirrer from Thermo Fisher Scientific, but any similar instrument with sufficient surface on which to place the experimental setup will work.
  p. Turn off power supply and remove electrical leads when dye front reaches reference line at bottom of gels.
10. Semi-wet protein transfer (Troubleshooting).
  ***Note:*** Western transfer buffer should be prechilled to 4°C.
  a. Cut PVDF membrane to size of gel.
  b. In a large Petri dish, soak PVDF membrane in 100% methanol for 2 min.
  c. After methanol incubation, replace methanol with western transfer buffer for 15–20 min on a platform rocker.
  d. Similarly, soak filter paper and foam pads (from Mini Trans-Blot electrophoretic transfer cell) in western transfer buffer for 15–20 min.
  e. Remove tank lid from electrophoresis setup and lift out electrode assembly, pouring off and discarding running buffer within the inner buffer chamber.
  f. Open assembly arms and remove gel cassettes.
  g. Remove gels from gel cassettes by separating the two plates of the cassettes using provided spatula.
  h. Rinse gels briefly in water and, using a disposable Petri dish, equilibrate with 4°C western transfer buffer for 15 min.
  i. Place electrophoretic cassette, gray side down, on a flat, clean surface.
  j. Place one presoaked foam pad on the gray side of the cassette.
  k. Place filter paper sheet on foam pad.
  l. Carefully place equilibrated gel on top of filter paper.
  m. Place presoaked PVDF membrane on top of gel.
  n. Place a piece of filter paper on PVDF membrane.
  o. Complete the transfer sandwich by adding a second foam pad.
  p. Roll out any bubbles forming within the stack.
  q. Close the cassette without moving the transfer sandwich.
  r. Place cassette in module.
  s. Repeat for second gel, if applicable.
  t. Add frozen blue cooling unit.
  u. Fill the tank to the blotting mark with 4°C western transfer buffer.
  v. Add stir bar to tank, and place lid on unit.
  w. Plug electrical leads into power supply.
  x. Transfer protein to PVDF membranes for 8–12 h at 4°C on a magnetic stirrer using a power output of 30 V.
    This step checks for equal loading of protein and potential transfer defects (*e.g*., air bubbles trapped between PVDF membrane and gel).
11. Ponceau S staining.
  a. To remove the western transfer buffer from the PVDF membrane, wash the PVDF membrane in MilliQ H_2_O 3 times for 1 min each on a rocker.
  b. Immerse membrane in Ponceau S staining solution for 5–10 min on a rocker.
    ***Alternative***: Our laboratory uses a platform rocker from Corning, but any rocker with a sufficiently large, non-slip surface and variable speeds will suffice.
  c. Wash membrane in MilliQ H_2_O 2–3 times until desired staining intensity is achieved.
  d. Visualize staining with the ChemiDox XRS+ system.
12. Blocking.
  a. Place PVDF membrane in a fresh Petri dish.
  b. For detection of phosphorylated proteins (e.g., p-S6), block PVDF membrane with 3% BSA in TBST. For unphosphorylated proteins (e.g., S6 or β-actin), block PVDF membrane with 5% non-fat milk in TBST. Blocking in either scenario should occur on a rocker at 4°C for 1 h.
13. Incubation with primary antibody.
  a. After blocking, wash the PVDF membrane with TBST a minimum of 2 times for 5 min each on a rocker.
  b. After washing, incubate PVDF membrane with primary antibody diluted in TBST with 3% BSA or 5% non-fat milk, per Step 12b.
  c. Incubate PVDF membrane in primary antibody for 10 h at 4°C with continuous rocking.

***Note:*** Primary antibody incubation times are protocol- and antibody-dependent and likely require individual optimization.

14. Incubation with secondary antibody.
  a. Wash PVDF membrane 3 times with TBST for 5 min on rocker at 20°C–22°C.
  b. After washing, incubate PVDF membrane with secondary antibody specific to host of primary antibody, conjugated to HRP, diluted in TBST with either 3% BSA or 5% milk (per Step 12b), at 4°C for 1 h on a rocker.
  c. After incubation with secondary antibody, wash PVDF membrane 3–4 times with TBST for 5 min each at 20°C–22°C on a rocker to remove any unbound secondary antibody.
15. Substrate detection.
  a. Prepare working solution of equal parts SuperSignal West Pico PLUS Luminol/Enhancer Solution and Stable Peroxide Solution to cover surface of the membrane.
  b. Incubate PVDF membrane in working solution for 5 min at 20°C–22°C by gently shaking by hand to ensure surface of membrane is evenly covered.
  c. Remove PVDF membrane from working solution and place blot development folder over membrane.
  d. Image membrane using Image Lab software associated with the ChemiDoc XRS+ System.

***Alternative***: Our laboratory uses the ChemiDoc XRS+ System to visualize membranes, but any similar chemiluminescent imaging system and software will suffice.

16. Densitometry normalization and quantification of bands using ImageJ.
  a. To quantify signal emitted by a protein band, export membrane image from the ChemiDoc XRS+ System as a TIFF file.
  b. Open TIFF file in ImageJ.
  c. Use the rectangle tool to define the region of interest (ROI) around first lane.
  d. Press 1 to select rectangle box as first sample. Drag box to second sample and press 2 to select box as second sample.
  e. Press 3 to generate profile plot for ROIs.
  f. Use the straight-line tool to enclose peaks for each sample.
  g. Using the wand tool, click the inside of each peak to measure its area.
  h. Export the peak areas to statistical software (our laboratory uses GraphPad Prism) for further analysis.
  i. Normalize experimental samples (e.g., p-S6 and S6) to housekeeping protein (e.g., β-actin).
  j. Determine change in phosphorylation of S6 protein with lentiviral infection as: normalized p- S6/normalized S6.

## Expected outcomes

The use of *in vitro* intestinal enteroids as a model for the *in vivo* intestine has become prevalent due to the translational opportunities provided by these tissues.^25^ Using source tissue derived from preterm infants, these cultures can be applied to studies of nutritional absorption and ontogenetic nutrient sensing within the intestine. Lentiviral knockdown of *TSC2* serves as a model of mTORC1 overexpression, a state associated with epithelial differentiation^26^ and increased intestinal regeneration post-injury.^27^ We opted for lentiviral gene knockdown over alternative methods because lentiviruses transfect both dividing and senescent cells, resulting in relatively high transduction efficiency.^28^ Here, we provide images of optimal (Figure 1A) and suboptimal (Figure 1B) enteroid morphologies for transfection. In addition, we provide examples of experimental readouts to evaluate lentiviral transduction efficiency and explore the effects of lentiviral gene knockdown in enteroids. Figure 2B illustrates representative results of flow cytometry analysis of transduction efficiency and cell viability following lentiviral transfection, while Figure 2C highlights examples of western blots from transfected enteroid cell lysates. Quantification of western blot bands through densitometry is also demonstrated in Figure 2C. In addition to those techniques demonstrated here, qRT- PCR, enteroid growth assays, and ELISAs, for example, represent further downstream assays to evaluate the effects of enteroid *TSC2* knockdown.

## Limitations

This protocol utilizes donor tissues derived from intestinal surgical resections. Due to donor variability, enteroid phenotypes may differ in response to both lentiviral transfection and the resultant gene knockdown. During enteroid maintenance, our laboratory routinely uses antimicrobial agents to inhibit microbial and fungal contamination. However, we discontinue use of these compounds during experiments as they are known to alter cellular physiology.^29^ Therefore, investigators should be cognizant of increased risk for microbial contamination during experimental periods. As with all enteroid models, the cell types represented in these ‘mini intestines’ are only of epithelial lineage. Because these models exclude mucosal immune cells, nerves, muscle, and a functional microbiome, enteroids cannot truly be considered representative of *in vivo* physiology. Finally, a plethora of enteroid media variations have been described. As use of different media recipes will likely alter experimental results, enteroid media composition should be seriously considered when evaluating experimental variability.

## Troubleshooting

### Problem 1

Matrigel did not completely coat bottom of well, related to Preparation Step 1.

### Potential solution

• Ensure Matrigel and the plate in which Matrigel is deposited remain on ice during the entirety of the plating procedure.
• During Matrigel plating, aspirate ice-cold PBS from only one well at a time to ensure plate does not warm or dry during procedure.

### Problem 2

Enteroids do not dissociate well into a single-cell suspension, related to Preparation Step 2.

### Potential solution

• Leave enteroids in TrypLE Express in 37°C incubator for slightly longer. Monitor cells often to ensure cell viability does not suffer.
• After transferring well contents to a 15 mL centrifuge tube and mechanically disrupting enteroids 20–30 times with a pipette, pass cells through a 37 μm cell strainer (e.g., STEMCELL Technologies, 27215) to eliminate cell clumps.

### Problem 3

Cell viability is low, related to Preparation Step 3.

### Potential solution

• Perform manual (hemocytometer) counting to verify numbers from automated cell counter (e.g., Countess 3).
• Reduce incubation time in TrypLE Express.
• Ensure dead, unattached cells are removed via media change the day after plating, before completing second layer of Matrigel sandwich.
• Include 2.50 μM CHIR-99021, a GSK-3β inhibitor,^30^ in culture media when preparing single-cell suspension in order to increase viability of stem cells, in particular.^31^

### Problem 4

Enteroid transduction efficiency is low, related to Step 1.

### Potential solution

• MOI is suboptimal for vector/cell type. Evaluate transduction efficiency of higher MOIs using control, GFP-expressing particles.
• Lentiviral particles have degraded due to freeze/thaw cycles, thereby decreasing the functional titer. Always prepare lentiviral particles on ice and aliquot into small working volumes for storage at −80°C.
• Spinoculation speed was insufficient to ensure direct contact of enteroids/viral particles. Verify cells are localized at bottom of 96-well microplate after spinning.
• Puromycin concentration is ineffective. Redo puromycin trial and optimize for 100% cell death in uninfected cells.

### Problem 5

Cell fixation affects GFP fluorescence during flow cytometry because 10% NBF contains 4% formaldehyde, related to Step 4.

### Potential solution

• Use a low-formaldehyde (1%) buffer.

### Problem 6

Cell concentration is low for flow cytometry analysis, related to Step 5.

### Potential solution

• Cells stick to polystyrene tubes, so ensure polypropylene 5 mL tubes are used.
• Increase concentration of BSA in cell diluent when running flow cytometry to avoid cells sticking to tube walls.
• Include more wells/treatment to increase cell density.
• During all steps involving cell transfer, pipette tips and tubes can be pre-treated with 5% BSA in PBS to inhibit cell adherence to plasticware.

### Problem 7

Flow cytometry compensation is difficult when using fluorescent protein-expressing samples, related to Step 5.

### Potential solution

• Use either GFP compensation beads (matching the specific GFP expressed by lentiviral particles) or constitutively GFP-expressing cell line to standardize compensation of GFP expression generated by enteroid transfection.

### Problem 8

The top of the PVDF membrane sticks to the transfer gel, related to Step 10.

### Potential solution

• Saturate PVDF membranes for a longer period in transfer buffer to ensure membranes do not stick to the gel and the transfer of high molecular weight proteins of interest, in particular, is not disrupted.

## Resource availability

### Lead contact

Further information and requests for resources and reagents should be directed to and will be fulfilled by the lead contact, Kathryn Burge (Kathryn-Burge@ouhsc.edu).

### Technical contacts

Technical questions on executing this protocol should be directed to and will be answered by the technical contacts, Karni Moshal (Karni-Moshal@ouhsc.edu) and Adam Wilson (Adam-Wilson@ouhsc.edu).

### Materials availability

This study did not generate new unique reagents.

### Data and code availability

This study did not generate or analyze datasets or code.
